# Sex-Dimorphic Gut Microbiota Modulation by Icariin in Older Mice

**DOI:** 10.1093/geroni/igaf122.1851

**Published:** 2025-12-31

**Authors:** Xiaoang Li, Liping Duan

**Affiliations:** Peking University Third Hospital, Beijing, Beijing, China (People’s Republic); Peking University Third Hospital, Beijing, Beijing, China (People’s Republic)

## Abstract

This study investigates the sexually dimorphic effects of icariin—a bioactive flavonoid from Epimedium—on gut microbiota remodeling in older C57BL/6J mice, evaluate its impact on microbial diversity, structure, and metabolic functions, and analyzes the role of sex differences in its intervention effects. Through 16S rRNA sequencing (V3-V4, Illumina NovaSeq) and PICRUSt2-based metabolic prediction, we demonstrate that 15 days icariin treatment (100 mg/kg/day) preferentially restores youthful microbial profiles in older mice (24-month-old), with females exhibiting 25% greater α-diversity improvement (Shannon index, p < 0.05) and 40% closer β-diversity proximity to young controls (Bray-Curtis, p = 0.008) compared to males. Sex-stratified analysis revealed distinct taxonomic shifts: aged females showed Lactobacillus enrichment (4.1-fold) linked to estrogen metabolism, while males displayed Akkermansia amplification (3.9-fold, p = 0.012) associated with mucin degradation. Functionally, icariin upregulated steroid biosynthesis pathways 3.2-fold in females versus 1.8-fold in males (q < 0.05), correlating with phytoestrogen-mediated microbial regulation. These findings not only establish icariin as a sex-specific modulator of gut microbiota aging but also provide mechanistic insights into sexual dimorphism in host-microbiome interactions, advocating for gender-precise nutraceutical strategies to counteract age-related dysbiosis.

